# Antiretroviral Therapy for Control of the HIV-associated Tuberculosis Epidemic in Resource-Limited Settings

**DOI:** 10.1016/j.ccm.2009.08.010

**Published:** 2009-12

**Authors:** Stephen D. Lawn, Katharina Kranzer, Robin Wood

**Affiliations:** a The Desmond Tutu HIV Centre, Institute of Infectious Disease and Molecular Medicine, Faculty of Health Sciences, University of Cape Town, Anzio Road, Observatory 7925, Cape Town, South Africa; b Department of Infectious and Tropical Diseases, Clinical Research Unit, London School of Hygiene and Tropical Medicine, London, UK

**Keywords:** HIV, Tuberculosis, Antiretroviral, Disease control, Mortality

## Abstract

Great progress has been made over the past few years in HIV testing in patients who have tuberculosis (TB) and in the scale-up of antiretroviral therapy. More than 3 million people in resource-limited settings were estimated to have started antiretroviral therapy by the end of 2007 and 2 million of these were in sub-Saharan Africa. However, little is known about what impact this massive public health intervention will have on the HIV-associated TB epidemic or how antiretroviral therapy might be used to best effect TB control. This article provides an in-depth review of these issues.

## HIV-associated tuberculosis epidemic

Tuberculosis (TB) remains a major challenge to global public health and has proved particularly difficult to control in regions with high HIV prevalence. Of the estimated global burden of 9.3 million new TB cases in 2007, 1.37 million (14.8%) were associated with HIV.^1,2^ TB remains a major cause of mortality, with an estimated 1.3 million deaths among individuals who are not infected with HIV and a further 0.5 million TB deaths among people who have HIV. The latter accounted for almost 25% of global AIDS-related mortality.

**Fig. 1:**
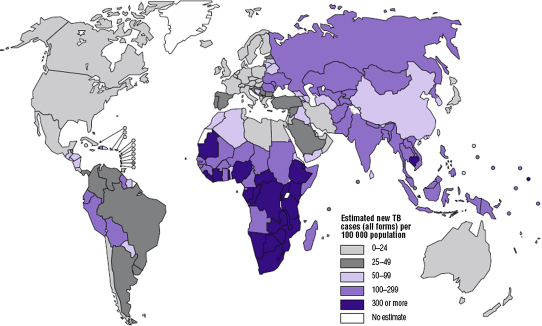
Estimated TB incidence rates by country for 2006. (*Reproduced from* World Health Organization. Global Tuberculosis Control. Surveillance, planning, and financing. WHO/HTM/TB/2008.393. Geneva (Switzerland): World Health Organization; 2008; with permission.)

HIV was a key factor underlying the approximately 1% annual increase in global TB incidence rates between 1990 and 2004. Sub-Saharan Africa has borne the brunt of this co-epidemic, accounting for 79% of cases of the global burden of HIV-associated TB in 2007. Here TB notification rates have increased three- to fivefold in many countries, especially in the south of the continent where HIV prevalence is highest. As a result, TB incidence rates in the region far exceed those observed in most other parts of the world (Fig. 1). Elsewhere, South and East Asia account for 11% of the global burden of HIV-associated TB and the combined epidemics of HIV and drug resistant TB are undermining TB control in Eastern Europe.^1^

## Millennium development goals for tuberculosis control

The Millennium Development Goals (MDG) TB control targets are to halt and start to reverse the rising incidence of TB and to halve the 1990 prevalence and death rates by 2015.^3^ Although the absolute number of TB cases occurring worldwide continued to increase in 2007, it was largely the result of global population growth. Global TB incidence rates are actually thought to have peaked in 2004 at 142 cases per 100,000 population and to have started to slowly decrease thereafter.^1,2^

This recent downward trend in global TB incidence was preceded by a sustained decrease in global TB prevalence since 1990.^1^ Rates of TB mortality (HIV-associated and non-HIV–associated) are also estimated to have peaked around the year 2000 and have since decreased.^1^ Despite these encouraging overall trends, however, the global MDG TB control target of halving the 1990 TB prevalence and death rates by 2015 will not be achieved with current rates of progress.^3^ Targets are projected to be missed in the regions of sub-Saharan Africa and Eastern Europe where the TB and HIV epidemics intersect. Concerted international public health action is needed.

## International public health response

Since the World Health Organization (WHO) declaration in 1993 that TB was a global emergency, the directly observed treatment, short-course (DOTS) strategy has served as the key public health intervention that has been widely implemented to effect global TB control.^4^ This strategy prioritizes the detection of patients who are sputum smear-positive presenting to health facilities with symptoms and aims to achieve high cure rates using short-course rifampicin-containing chemotherapy. Although this has been effective in most parts of the world, contributing to the sustained downward trend in global TB prevalence, it has nevertheless been comparatively ineffective in countries with high HIV prevalence.^1,2,5,6^ Although the DOTS strategy remains the foundation for TB control programs in high HIV-prevalence settings, it is clear that additional interventions are needed.

Under the leadership of the WHO and the Stop-TB Partnership, TB-HIV guidelines,^7^ a TB-HIV strategic framework,^8^ and an interim TB-HIV policy^9^ were published between 2002 to 2004 to address the challenge of HIV-associated TB in severely affected countries. The aim of these interventions is to reduce the burden of TB in people who are HIV-infected through use of TB prevention strategies, such as isoniazid preventive therapy, intensified case finding, infection control, and antiretroviral therapy (ART). A further aim is to reduce the impact of HIV in patients who have TB through HIV testing and use of trimethoprim-sulfamethoxazole prophylaxis and ART.

Great progress has been made over the past few years in HIV testing in patients who have TB^1^ and in the scale-up of ART.^10^ More than 3 million people in resource-limited settings were estimated to have started ART by the end of 2007 and 2 million of these were in sub-Saharan Africa.^10^ However, little is known about what impact this massive public health intervention will have on the HIV-associated TB epidemic or how ART might be used to best effect TB control. This article provides an in-depth review of these issues.

## Tuberculosis incidence rates in antiretroviral treatment cohorts

Data from 12 observational studies that included over 32,000 participants living in high-income countries (seven studies) and resource-limited countries (five studies) are shown in Table 1. In two of these studies, TB rates were simply compared before and after 1995 when triple-drug ART was introduced, whereas in the remaining studies TB rates were determined according to person-time of exposure to ART. All but two of these studies found a statistically significant reduction in TB risk during ART.

**Fig. 2:**
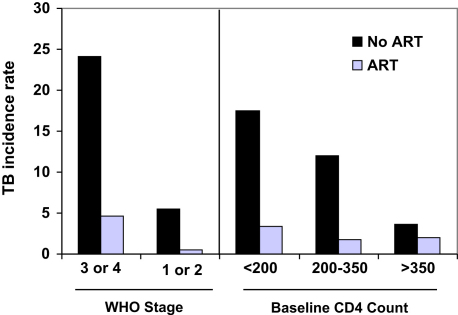
TB incidence (cases per 100 person-years) among patients who were HIV-infected in Cape Town, South Africa, who were or were not receiving antiretroviral therapy. Patients were stratified according to baseline CD4 cell count and WHO stage of disease. Overall, TB rates were approximately 80% lower among those receiving ART, which was observed across a broad spectrum of baseline immunodeficiency. (*Data from* Badri M, Wilson D, Wood R. Effect of highly active antiretroviral therapy on incidence of tuberculosis in South Africa: a cohort study. Lancet 2002;359(9323):2059–64).

In nine studies, the reductions in TB risk associated with use of antiretrovirals are expressed as hazard ratios adjusted for covariates, such as baseline patient characteristics and antiretroviral regimen. Triple-drug ART was associated with a risk reduction of more than 70% in the majority of these studies (n = 6) with a range of 54% to 92% (see Table 1). This protective effect against TB was seen in countries with low-TB burden, such as the United States,^11^ and South Africa,^12^ which has the highest burden of HIV-associated TB in the world.^2^ The benefits of ART are seen across a broad range of degrees of baseline immunodeficiency and clinical stage of disease, although the absolute reduction in TB rates is greatest in those with the most advanced HIV disease (Fig. 2).

**Fig. 3:**
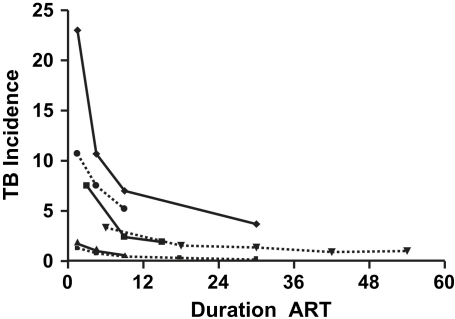
TB incidence rates during ART. The graph shows data from studies included in (see Table 2) in which changing TB incidence rates were calculated according to increasing duration of ART. The two lowest curves present data from studies conducted in high-income countries.^15,18^ The remaining four studies are from South Africa (diamonds^13^ and inverted triangles^14^), a range of resource-limited countries (circles^15^), and Uganda (squares^16^).

TB incidence rates in 14 ART cohorts are shown in Table 2. The observed rates are heterogeneous and are likely to vary according to the local TB incidence rates, the baseline degree of immunodeficiency, and the duration of ART. Several studies report rates stratified according to duration of ART and these are shown in Fig. 3. The figure illustrates the major differences between TB rates in various settings and the rapidity of the beneficial impact of ART. All of these studies show time-dependent reductions in TB rates, with most of the benefit occurring within the first 2 years of ART.^13–19^ Although reductions in TB incidence in high- and low-TB burden settings are proportionately similar,^15^ the greatest absolute reductions in TB risk of course occur in high-TB prevalence settings.

**Fig. 4:**
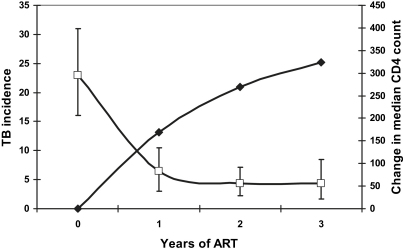
Decreasing TB incidence rates (cases/100 person-years, white squares) and rising median CD4 cell counts (cells/μL, black diamonds) during the first 3 years of ART. These data are from a community-based ART cohort in a township in Cape Town, South Africa. (*Data from* Refs.^13,19,56^).

**Fig. 5:**
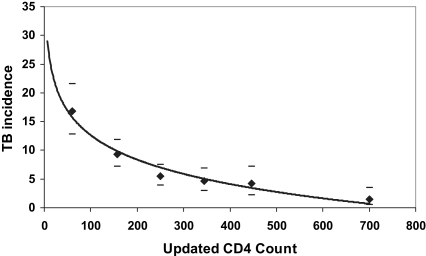
Relationship between updated CD4 cell-count measurements made every 4 months during 4.5 years of ART in a treatment cohort in a township in Cape Town, South Africa. Observed rates are shown as diamonds together with 95% confidence intervals indicated by bars. A logarithmic trend line is overlaid (R^2^ = 0.97). TB incidence rates are seen to fall substantially as CD4 cell counts increase during ART. (*Data from* Lawn SD, Myer L, Edwards D, et al. Short-term and long-term risk of tuberculosis associated with CD4 cell recovery during antiretroviral therapy in South Africa. AIDS 2009;23(13):1717–25.)

Baseline risk factors for incident TB during ART in resource-limited settings included low-CD4 cell count,^12–15^ advanced WHO stage of disease,^12,14^ younger age,^14,15^ low socioeconomic status,^12^ past history of TB,^20^ and male sex.^15^ In high-income countries, baseline risk factors for TB included low-baseline CD4 count and HIV transmission category.^15,18^ However, the risk of TB decreased as CD4 cell counts rose (Fig. 4). Thus, with increasing duration of treatment, the immunologic response to ART, rather than baseline patients' characteristics, emerges as the dominant predictor of TB risk.^13^ The strong relationship between TB rates and updated CD4 cell counts during ART is shown in Fig. 5.^19^

Data are scarce concerning TB rates among those with optimum immune recovery. However, in a study in South Africa, subjects achieving CD4 cell counts of greater than 500 cells/μL retained a TB rate that was approximately two-fold higher than that in subjects who were non-HIV–infected living in the same community.^19^ These data suggest that recovery of TB-specific immune function during ART may be incomplete even at high CD4 cell counts.

## Antiretrovirals and tuberculosis incidence at a community level

Despite the extensive data that have arisen from observational cohort studies, empiric data regarding the impact of ART on TB incidence rates at the community level are lacking. This is a difficult issue to study. In-depth observational studies of sentinel communities with high TB and HIV prevalence during scale-up of ART are needed. Outcome measures should include the impact of ART scale-up on TB incidence and prevalence, TB transmission, and TB-associated mortality. However, trends in TB incidence and mortality over time will inevitably be confounded by other variables, such as the natural evolution of the HIV epidemic, changes over time in socio-economic factors, and the efficiency of TB control programs. Ecological studies comparing settings differing in the rapidity and coverage of ART scale-up may provide further insights.

## Likely limited impact of antiretrovirals at community level

Despite the major beneficial impact of ART on TB rates in observational cohorts, mathematical modeling studies suggest that the impact of ART on TB rates at a population level will be limited.^21^ Various observations suggest that this conclusion is likely to be true^22^ and are discussed in full in Box 1.

### Risk of Tuberculosis Before Starting Antiretroviral Treatment

Following HIV seroconversion, risk of TB doubles and continues to increase as CD4 cell counts decline.^12,23–25^ Patients, therefore, typically remain at a high risk of TB for many years before eligibility for ART. Further compounding this, HIV infection is often only diagnosed once patients present to medical services with advanced immunodeficiency and either have active TB or high risk of developing TB in the short term.^26^

In addition to TB occurring before enrollment for ART, a substantial burden of prevalent TB may be diagnosed in ART services during screening before starting treatment.^13,16,27,28^ The proportion detected may vary according to the intensity of investigation and availability of mycobacterial culture. In two studies from South Africa, 20% to 25% of adults referred for ART who did not already have a TB diagnosis were found to have sputum culture-positive TB, the majority of which was smear-negative.^27,28^

The overall cumulative risk of TB before initiating ART may therefore be very high. In one ART service in Cape Town, this represented two thirds of adult patients.^13^ Thus, ART is frequently implemented too late in the course of HIV progression to prevent much of the overall burden of HIV-associated TB.

### Incidence of Tuberculosis During Antiretroviral Therapy

In addition to TB occurring pre-ART, TB incidence rates during ART persist at rates substantially higher than background (see Table 2). During the initial months of treatment, a proportion of TB cases present as a result of the unmasking of subclinical TB during ART-induced restoration of TB-specific immune responses.^19,29,30^ Through this mechanism, the risk of TB during ART may actually increase in the short term after starting ART. However, the size of this effect will be highly dependent on the efficiency with which patients are screened for clinical and subclinical TB before commencing ART. Unmasking TB was estimated to account for more than one third of the TB cases presenting during the first 4 months of ART in a treatment service in South Africa.^19^

In the longer term, TB rates during ART appear to be largely dependent on changes in the CD4 cell count over time. With CD4 cell counts less than 200 cells/μL, TB rates exceeded 9.0 cases/100 person-years (PY) in the same study from South Africa.^19^ Rates at higher CD4 counts were substantially lower, but nevertheless remained more than five-fold higher than rates in individuals who were non-HIV-infected living in the same community.^13,19^

Persistence of high TB rates in ART cohorts is likely to reflect the fact that most patients spend prolonged periods at CD4 cell counts less than 500 cells/μL. In addition, immunologic studies also suggest that defects in functional TB-specific immune responses may persist despite ART,^31^ leaving patients vulnerable to TB long-term. This may be compounded by high rates of nosocomial exposure to TB within overcrowded ART services where infection control procedures are often lacking.^32,33^

Despite high early mortality risk,^34^ long-term survival after the first year of ART is likely to be good in patients who are in treatment programs in sub-Saharan Africa. In light of the persistence of high rates of TB during ART, the life-time cumulative risk of TB in survivors will inevitably be high. It is not yet clear whether this is reduced compared with the cumulative life-time risk in patients who are HIV-infected in the pre-ART era.

A further critical factor affecting the magnitude of the impact of ART on community TB rates is the coverage of ART. In mathematical modeling studies, high coverage with ART was needed to impact TB rates.^21^ However, coverage in sub-Saharan Africa was only approximately one third of those estimated to be in need at the end of 2007.^10^ In most countries, rates of HIV-testing are low and only a small proportion of individuals who are HIV-infected know their HIV status.

The magnitude of any impact of ART on TB transmission is unknown. It has been generally assumed that patients who are HIV-infected are not the key drivers of TB transmission in the community in view of their comparatively low infectivity. If true then ART is unlikely to have a major impact on community TB transmission. Conversely, however, patients receiving ART survive longer and remain at substantial risk of TB that may be more likely to be sputum smear-positive as immune function improves. However, transmission risk among such patients remains undefined.

Perhaps the most important issue relating to TB transmission is that occurring between individuals who are HIV-infected in health care settings.^33^ This issue was exemplified by the outbreak of multidrug-resistant and extensively drug-resistant TB in a hospital-based ART service in rural South Africa from 2005 to 2006.^32^ Although ART may serve as a potent preventive therapy for TB, ART services without adequate infection-control measures may nevertheless be sites associated with high risk of nosocomial TB transmission and outbreaks.

## Enhancing the impact of antiretrovirals on tuberculosis control

### Earlier Initiation of Antiretroviral Therapy

It is likely that ART could be used much more effectively as a TB-prevention strategy (Box 2). ART should be initiated much earlier in the course of disease than is currently being done. In sub-Saharan Africa, the median CD4 cell count at baseline in ART programs is often in the range of 100 to 150 cells/μL^34^ and many patients have already had TB before starting ART. Earlier HIV diagnosis would permit more timely initiation of ART, thereby enhancing its role as a preventive intervention. Consideration should be given to revision of ART eligibility guidelines in countries, such as South Africa, where treatment is restricted to patients who have WHO stage 4 disease or blood CD4 cell counts of less than 200 cells/μL.^35^ A randomized controlled trial of early (CD4 200–350 cells/μL) versus late (CD4 <200 cells/μL) initiation of ART in Haiti was discontinued in 2009 following interim analysis.^36^ In the delayed initiation group, the mortality was fourfold higher and the TB incidence rate was twofold higher than in the early initiation group, clearly demonstrating the benefits to be derived from earlier treatment.

A mathematical modeling study explored the potential impact of a test and treat strategy whereby all patients who are diagnosed with HIV are immediately offered ART irrespective of the blood CD4 cell count.^38^ Although interest has primarily focused on the potential of such a strategy for HIV prevention, this also has the potential to impact TB control. The rapidity of scale-up of ART may also be an important variable that affects not only the numbers of deaths averted by ART^39^ but also the number of TB cases averted.

### Adjunctive Interventions

Because TB rates do not decrease to background levels during long-term ART, use of concurrent adjunctive interventions is needed. Three key interventions are encompassed within the WHO 3I's strategy launched in April 2008.^37^ This three-pronged strategy includes intensified case finding, isoniazid preventive therapy, and infection control.

Intensified case finding is needed in ART services as this designates patients as either having active TB (and in need of treatment) or being TB-free and potentially benefitting from isoniazid preventive therapy. Initiation of TB treatment in those who have disease also plays a critical role in reducing the risk of nosocomial TB transmission. Observational data have examined the potential role of isoniazid preventive therapy concurrently with ART in Brazil. Although isoniazid alone was not associated with significant reductions in TB risk compared with the nonintervention group, it was suggested that concurrent isoniazid preventive therapy during ART provided an additive benefit.^40^ However, this requires confirmation by randomized controlled trials.

## Impact of antiretrovirals on mortality risk

TB case fatality rates (the proportion of patients dying while receiving antituberculosis treatment) in Africa are 16% to 35% among patients who are infected with HIV and not receiving ART and 4% to 9% among individuals who were not infected with HIV.^41^ The authors identified observational studies (n = 8) that provided information on the impact of ART on survival of patients who have HIV-associated TB (Table 3). Of these, half were from high-income countries and half were from resource-limited settings.

One study using national data from The Netherlands found a 54% reduction in risk of death of patients who had HIV-associated TB treated in the ART era compared with in the pre-ART era.^42^ Of seven further studies, six found that use of ART was associated with significant reductions in mortality risk of 64% to 95% in adjusted analyses (see Table 3). The remaining study from Malawi found no such association but analyses were not adjusted for CD4 cell counts and ART was not commenced during the intensive phase of TB treatment when most deaths occurred.^43^

A key issue that remains to be resolved regarding the clinical management of patients who have HIV-associated TB is the optimal time to start ART during TB treatment. Early mortality rates are extremely high among patients waiting to start ART in resource-limited settings.^44,45^ However, early initiation of ART in those who have HIV-associated TB increases the risk of TB immune reconstitution disease.^46^ This increase is associated with mortality risk in a small proportion of patients^46,47^ but may be greater in those who have neurologic involvement.^48^ Overall, the risk/benefit ratio is likely to favor initiation of ART in the initial weeks of TB treatment, but the outcome of randomized controlled trials are awaited.

## Antiretrovirals and tuberculosis treatment outcomes

Data concerning the impact of ART on TB treatment outcomes other than death are scarce. A large retrospective observational cohort study in San Francisco reported that use of ART was associated with significant shortening of the time to smear and culture conversion.^49^ A systematic review of 32 studies^50^ and recently published data from Rio de Janeiro, South African gold mines, and San Francisco^49,51,52^ confirm that patients who have HIV-associated TB are at increased risk of recurrent disease, especially those who have low CD4 cell counts. Various strategies can be used to address high recurrence rates, including prolongation of the duration of TB therapy^49,53^ and use of secondary isoniazid prophylaxis.^54,55^ However, more recent data from a study in Brazil have now shown that recurrence rates were halved in patients who had TB who received ART.^51^ It remains to be determined whether combining ART and isoniazid has an additive effect.

## Summary

The HIV-associated TB epidemic is undermining progress toward TB control and additional interventions must be used in combination with the DOTS strategy in resource-limited settings. Observational cohort studies in a wide range of settings have demonstrated that the use of ART is associated with a 54% to 92% reduction in TB incidence rates and a halving of the risk of TB recurrence. ART is also a potent means of improving the survival of those who have HIV-associated TB, reducing mortality rates by 64% to 95%. Thus, ART confers huge benefits to individual patients.

However, limited data are available concerning the effects of ART on TB rates at a population level. Mathematical modeling and a range of empiric observations suggest that this may be limited, largely because of the high burden of TB that occurs before ART initiation and the persistence of TB rates several-fold higher than background rates during long-term ART. Thus, ART is likely to have limited impact on lifetime cumulative risk of TB. To enhance the impact on community TB control, it is likely that ART will need to be implemented with high-population coverage and at much higher CD4 cell counts than is currently the case. Adjunctive interventions, such as those included in the 3I's policy, may help reduce rates further.

Despite the likely limited impact on TB rates at a community level, ART nevertheless transforms the prognosis of patients who have HIV-associated TB. By reducing mortality rates, ongoing ART scale-up may accelerate progress toward the MDG TB control target of halving the 1990 mortality rate by 2015.

## Figures and Tables

**Table 1:** Studies (n = 12) reporting the impact of antiretroviral therapy on tuberculosis incidence rates in observational cohorts

Study	Setting	N	Study Period	Study Design	Impact of ART on TB Incidence Rates	Adjusted Hazards Ratio (95%CI)
*Studies comparing TB rates in cohorts before and after introduction of ART*						
Brodt et al, 1997^57^	Germany	1003	1992–1996	Cohort of homosexual men 1992–1996	No change in overall cohort incidence rates (range, 2.1–2.7 cases/100 PY)	–
Kirk et al, 2000^58^	Europe	6,972	1994–1999	EuroSIDA multicenter cohort 1994–1999	Overall rate in cohort decreased from 1.8 cases/100 PY to 0.3 cases/100 PY	–
*Studies comparing TB rates in patients receiving or not receiving ART*						
Ledergerber et al, 1999^17^	Switzerland	2410	1995–1997	Swiss HIV Cohort Study	Rate 0.78 cases/100 PY pre-ART and 0.22 cases/100 PY during first 15 months ART	–
Jones et al, 2000^11^	United States	–	1992–1998	Multicenter cohort Adult/Adolescent Spectrum of HIV Disease project	Steep decreases in TB incidence rates	0.2 (0.1–0.5)
Girardi et al, 2000^59^	Italy	1360	1995–1996	Multicenter cohort	Not stated	0.08 (0.01–0.88)
Santoro-Lopes et al, 2002^60^	Brazil	255	1991–1998	Prospective cohort	Not stated	0.2 (0.04–1.13)
Badri et al, 2002^12^	South Africa	1034	1992–2001	Rates compared in separate prospective observational cohorts receiving or not receiving ART	Markedly lower TB rates across a broad spectrum of baseline CD4 counts and WHO stage	0.19 (0.09–0.38)
Golub et al, 2007^40^	Brazil	11,026	2003–2005	Multicenter retrospective cohort	Rates among those receiving and not receiving ART were 1.9 and 4.0 cases/100 PY, respectively	0.46 (0.33–0.63)
Miranda et al, 2007^61^	Brazil	463	1995–2001	Multicenter retrospective study	Rates among those receiving and not receiving ART were 1.2 and 13.4 cases/100 PY, respectively	0.2 (0.1–0.6)
Muga et al, 2007^62^	Spain	2238	1980s–2004	Multicenter seroconverter cohort	Marked reduction in rates after 1995 in all HIV transmission categories	0.31 (0.17–0.54)
Moreno et al, 2008^63^	Spain	4268	1997–2003	Multicenter hospital-based cohort	Rates among those receiving and not receiving ART were 0.5 and 1.6 cases/100 PY, respectively	0.26 (0.16–0.40)
Golub et al, 2009^64^	South Africa	2778	2003–2007	Retrospective data from two study sites	Rates among those receiving and not receiving ART were 4.6 and 7.1 cases/100 PY, respectively	0.36 (0.25–0.51)

*Abbreviation:* PY, person-years.

**Table 2:** Studies (n=14) reporting tuberculosis incidence rates during antiretroviral therapy

Study	Setting	N	Median / Mean Follow-up (Months)	Median Baseline CD4 Cell Count (Cells/μL)	TB Cncidence, Cases/100 PY (Months of ART)	Estimated National TB Incidence Rate (Per 100 Population)^a^
High-income countries						
Girardi et al, 2005^18^	Germany, Switzerland, France, Netherland, UK, Canada, United States	17,142	25.8	280	1.31 (0–3) 0.78 (4–6) 0.46 (7–12) 0.33 (13–24) 0.15 (25–36)	0.005–0.016
Brinkhof et al, 2007^15^	Europe, North America	22,217	11.0	234	1.7 (0–3) 1.0 (4–6) 0.6 (7–12)	<0.015
Moreno et al, 2008^63^	Spain	4268	46.0	324	0.5	0.035
Resource-limited settings						
Badri et al, 2002^12^	South Africa	1034	16.8	254	2.4	0.406
Santoro-Lopes et al, 2002^60^	Brazil	284	22.0	–	8.4	0.071
Lawn et al. 2005^14^	South Africa	346	40.0	242	3.35 (0–12) 1.56 (13–24) 1.36 (25–36) 0.90 (37–48) 1.01 (49–60)	0.576
Seyler et al, 2005^20^	Côte d'Ivoire	129	26.0	125	4.8	0.368
Lawn et al, 2006^13^	South Africa	1002	0.9	96	23.0 (0–3)10.7 (4–6) 7.0 (7–12) 3.7 (13–24)	0.898
Bonnet et al, 2006^65^	Kenya Malawi Cameroon Thailand Cambodia	3151	3.7 6.7 11.1 3.7 7.3	–	17.6 14.3 4.8 10.4 7.6	0.419 0.416 0.194 0.142 –
Golub et al, 2007^40,64^	Brazil	11,026	17.0	–	1.90	0.053
Miranda et al, 2007^61^	Brazil	245	–	–	1.2	0.064
Brinkhof et al, 2007^15^	Botswana, Brazil, Côte d'Ivoire, India, Kenya, Nigeria, Malawi, Morocco, Senegal, South Africa, Thailand, Uganda	4540	9.6	107	10.7 (0–3) 7.5 (4–6) 5.2 (7–12)	0.055–0.852
Moore et al, 2007^16^	Uganda	1044	17.0	127	3.9 (overall) 7.5 (0–6) 2.4 (7–12) 1.9 (13–18)	0.385
Walters et al, 2008^66^	South Africa	290 (pediatric)	–	–	6.4	0.898

*Abbreviation:* PY, person-years.

a National TB incidence estimates at the midpoint of the study duration; data sourced from World Health Organization. Global tuberculosis control: epidemiology, strategy, financing. Geneva (Switzerland): World Health Organization; 2009. WHO/HTM/TB/2009.411.

**Table 3:** Observational cohort studies (n=8) showing the impact of antiretroviral therapy on mortality among patients who have HIV-associated tuberculosis

Study	Country	Study Design	Outcome
Dheda et al, 2004^67^	United Kingdom	Retrospective study of patients who had HIV-TB (n = 99) treated in pre-ART era and in ART era	Adjusted hazards of death or new AIDS-defining illness was 0.34 (95%CI, 0.18–0.63) during ART era
Manosuthi et al, 2006^68^	Thailand	Retrospective cohort study (n = 1003) comparing mortality in a historic natural- history cohort with rates in an ART cohort	The adjusted hazards of death associated with use of ART was 0.05 (95%CI, 0.02–0.12).
Akksilp et al, 2007^69^	Thailand	Prospective cohort (n = 329) comparing patients receiving and not receiving ART	Adjusted hazards of death was 0.2 (95%CI, 0.1–0.4)
Zachariah et al, 2007^43^	Malawi	Retrospective observational cohort in which a proportion of patients started ART during the continuation phase of TB treatment (n = 658)	No difference in mortality between patients who chose or did not choose to receive ART, but potential allocation bias according to degree of immunodeficiency and most deaths occurred pre-ART during intensive phase
Nahid et al, 2007^49^	United States	Retrospective observational cohort (n = 264) 1990–2001 spanning pre-ART and ART era	Use of ART protected against mortality compared with patients who did not receive ART (hazard ratio 0.36, 95% CI 0.14–0.91)
Haar et al, 2007^42^	Netherlands	Retrospective observational study of national data 1993–2001 spanning pre-ART and ART era	Compared with 1993–1995, adjusted odds of death during 1999–2001 was 0.46 (95%CI, 0.24–0.89), whereas no such change was observed among patients who had TB and were not infected with HIV
Varma et al, 2009^70^	Thailand	Prospective multicenter observational study (n = 667) comparing patients receiving and not receiving ART	Adjusted hazards of death among those who received ART was 0.16 (95%CI, 0.07–0.36)
Velasco et al, 2009^71^	Spain	Retrospective observational cohort 1987–2004 (n = 313) comparing patients receiving and not receiving ART	Compared with no ART, initiation of ART within the first 2 months of TB treatment was associated with an adjusted hazards of death of 0..37 (95%CI, 0.17–0.66)

## Box 1 Reasons why scale-up of antiretroviral therapy is likely to have only limited impact on tuberculosis incidence rates at the community level

• Much HIV-associated TB occurs before initiation of ART
• High rates of TB persist during ART
• Prolonged survival of patients on ART is associated with a high life-time cumulative risk of TB
• Limited coverage of ART in the community and limited compliance with treatment
• Impact of ART on TB transmission in the community is suspected to be low

## Box 2 Potential ways to enhance the impact of antiretroviral therapy on tuberculosis prevention at a community level

• Diagnose HIV earlier and initiate ART at higher CD4 cell counts
• Increase coverage of ART in the community
• Increase rapidity of scale-up of ART
• Use of adjunctive interventions with ART, such as those included within the WHO 3I's strategy (intensified case finding, isoniazid preventive therapy and infection control)^37^
